# Enhancing Mesenchymal Stromal Cell Potency: Inflammatory Licensing *via* Mechanotransduction

**DOI:** 10.3389/fimmu.2022.874698

**Published:** 2022-07-06

**Authors:** Max A. Skibber, Scott D. Olson, Karthik S. Prabhakara, Brijesh S. Gill, Charles S. Cox

**Affiliations:** ^1^ Department of Pediatric Surgery, McGovern Medical School, University of Texas Health Science Center at Houston, Houston, TX, United States; ^2^ Department of Surgery, McGovern Medical School, University of Texas Health Science Center At Houston, Houston, TX, United States

**Keywords:** mesenchymal stem cell, mechanotranduction, bioreactor, immunomodulation, inflammatory licensing, wall shear stress, biomechanical culture, parallel-plate bioreactor

## Abstract

Mesenchymal stromal cells (MSC) undergo functional maturation upon their migration from bone marrow and introduction to a site of injury. This inflammatory licensing leads to heightened immune regulation *via* cell-to-cell interaction and the secretion of immunomodulatory molecules, such as anti-inflammatory mediators and antioxidants. Pro-inflammatory cytokines are a recognized catalyst of inflammatory licensing; however, biomechanical forces, such as fluid shear stress, are a second, distinct class of stimuli that incite functional maturation. Here we show mechanotransduction, achieved by exposing MSC to various grades of wall shear stress (WSS) within a scalable conditioning platform, enhances the immunomodulatory potential of MSC independent of classical pro-inflammatory cytokines. A dose-dependent effect of WSS on potency is evidenced by production of prostaglandin E2 (PGE_2_) and indoleamine 2,3 dioxygenase 1 (IDO1), as well as suppression of tumor necrosis factor-α (TNF- α) and interferon-γ (IFN-γ) production by activated immune cells. Consistent, reproducible licensing is demonstrated in adipose tissue and bone marrow human derived MSC without significant impact on cell viability, cellular yield, or identity. Transcriptome analysis of WSS-conditioned BM-MSC elucidates the broader phenotypic implications on the differential expression of immunomodulatory factors. These results suggest mechanotransduction as a viable, scalable pre-conditioning alternative to pro-inflammatory cytokines. Enhancing the immunomodulatory capacity of MSC *via* biomechanical conditioning represents a novel cell therapy manufacturing approach.

## Introduction

Mesenchymal stromal cell (MSC) anti-inflammatory function and role in injury resolution have led to their therapeutic application for a number of indications characterized by pathological inflammation. MSC are thought to mobilize to a site of injury (1, 2) and engage in paracrine crosstalk with immune effector cells through cell-cell contact and locally secreted factors (3). These interactions result in broad downstream effects often suppressing cytokine production, proliferation, chemotaxis, and skewing differentiation of different immune cells to anti-inflammatory phenotypes (2, 4–6). Despite early promise in pre-clinical studies and some initial clinical trials, many studies have struggled to demonstrate significant efficacy at scale. This is likely due to the interaction of many factors, including donor heterogeneity, lot-to-lot variability in MSC preparations, limited predictive value of existing disease-specific potency markers, an incomplete understanding of critical characteristics that dictate clinical benefit, and the dynamic nature of many MSC applications, particularly when used to treat acute injury and trauma (3, 7–9).

In an effort to improve their efficacy, some studies manipulate MSC in culture to simulate inflammatory licensing (1, 10–12). This strategy aims to mimic conditions after injury when endogenous MSC are exposed to various damage signals and inflammatory cues. Anti-inflammatory changes to the MSC secretome have been reported following *in-vitro* exposure to pro-inflammatory cytokines, such as interleukin-1 beta (IL-1β) (13–15), tumor necrosis factor alpha (TNF-α) (4, 16), interferon gamma (IFN-γ) (4, 17, 18), lipopolysaccharide (LPS) (19, 20), and Polyinosinic:polycytidylic acid (Poly I:C) (21, 22). Not wholly anti-inflammatory, the MSC response elicited by these cytokines can include classical pro-inflammatory signals, such as chemokines, metalloproteins, and TNF-α (4, 10, 12, 13, 22). Other priming studies have employed culture manipulations to pre-activate MSC including the use of hypoxia (23, 24) and low serum (25, 26). While promising, these approaches carry additional associated costs as well as down-stream complications to large-scale cell manufacturing regulatory approval (5, 8, 12). When resolved, primed MSC attained by *in vitro* licensing are often considered the next generation of MSC therapies to treat acute and sub-acute inflammation-associated injuries, such as traumatic brain injury (TBI) and ischemic stroke (3, 7, 9, 27).

*In-vitro* mechanotransduction, replicating the wall shear stress (WSS) exposure that MSC might withstand during development or after injury-induced migration, exists as a novel pre-conditioning technique (28–30). Biomechanical cues, such as WSS, have demonstrated profound effects on MSC immunomodulatory potential and paracrine signaling (28). Various magnitudes of WSS exist throughout vasculature and different shear stress patterns are reported to have distinct implications on MSC phenotype and secretome profiles (31). Both, the bone marrow and lymphoid tissues where MSC might dwell or become arrested after egress place MSC at a solid-fluid interface. From this boundary, MSC not only engage in molecular crosstalk but react to extrinsic, biophysical cues by altering transcriptional patterns (6, 32–35). Notably, our group found that a three-hour exposure to 15 dyne/cm^2^ strongly promoted the immune regulatory function of five human bone marrow MSC (hBM-MSC) cell lines. This was evidenced by increased transcription of *PTGS2, HMOX1, NFκB, IL1RN*, and *TGFβ1/2*, secretion of prostaglandin E_2_ (PGE_2_), and suppression of TNF-α production in a splenocyte co-culture (28).

The current study builds on our previous work by investigating how a range of physiologic WSS magnitudes affect the prospective cell therapy’s clinical translatability in a bioreactor scaled up for research purposes. Here we demonstrate a dose-dependence of mesenchymal stromal cells’ anti-inflammatory potential on WSS magnitude in a scalable system. Using a novel parallel-plate bioreactor (PPB) designed for accurate WSS application, we exposed adherent human adipose tissue derived MSC (hAD-MSC) and hBM-MSC to shear stress magnitudes of 0, 4, 8, and 12 dyne/cm^2^. The mechanotransduced MSC (WSS-MSC) were compared to MSC grown in conventional tissue culture flasks (Static-MSC) by viability, cellular yield after WSS exposure, PGE_2_ production, and indoleamine 2,3 dioxygenase 1 (IDO1) production criteria. The optimal WSS magnitude (8 dyne/cm^2^) was selected and further studied using a cGMP-compliant hBM-MSC product, confirming that WSS-MSC reduced inflammatory cytokine secretion by activated splenocytes. The effects of WSS on the transcriptome were then evaluated using RNA-seq. Optimizing fluid shear stress magnitude by assessing a matrix of viability, yield, identity, and immunomodulatory potential represent the initial considerations of translating a mechanotransduction-based preconditioning strategy for clinical applications.

## Materials and Methods

### Cell Culture

MSC culturing was carried out according to previously published efforts (28, 36, 37). Xeno-free human bone marrow MSC (hBM-MSC) were obtained from RoosterBio, Inc (Frederick, MD), specifically from their cGMP-simulated cell bank mirroring their cGMP-compliant products. The hBM-MSC acquired were from a single donor. The hBM-MSC were expanded according to manufacturer’s suggestion in RoosterNourish-MSC-XF medium. Once cultures had reached 70% confluency, RoosterNourish-MSC-XF medium was removed, the cells were washed with phosphate-buffered saline (PBS; Life Technologies), and the adherent cells population was harvested with TrypLE Express (Gibco, Grand Island, NJ) for five minutes at 37°C. Then, 10^6^ cells/mL aliquots were frozen in Cryostor CS10 (STEMCELL Technologies, Cambridge, MA) and representative aliquots were characterized according to previously published protocols (36). A single hBM-MSC cell line was utilized throughout the entirety of this investigation.

Human adipose tissue MSC (hAD-MSC) from a single donor were isolated according to a previously described methodology (37). To isolate hAD-MSC, subcutaneous adipose tissue samples were repeatedly washed with α-MEM (Life Technologies, Grand Island, NY) containing 50 μg/mL gentamicin and minced into 5 mm pieces. The samples were digested using a buffer of α-MEM, 300 IU/mL Collagenase Type II (Worthington Biochemicals, Lakewood, NJ), 1% bovine serum albumin (Gibco, Grand Island, NJ), and 50 μg/mL gentamicin for 55 minutes in a standard incubator environment. The liberated cells were then resuspended and expanded in a complete culture medium (CCM) consisting of α-MEM, 5% Stemulate human platelet lysate (hPL; Cook Regentec, Indianapolis, IN), 1% Glutamax (Gibco, Waltham, MA), and 10 μg/mL gentamicin (Gibco). Cultures were grown at 37°C/5% CO2 and media replenished every three days until 70% confluency was reached and subcultured to passage 3 (PDL 16.2). The adherent cells were harvested with TrypLE Express and frozen at 10^6^ cells per mL in Cryostor CS10 for future experiments.

### Computational Fluid Dynamics Studies

Individual components of the PPB, including plates, gaskets, and flow ports, were designed and assembled using the computer-aided design software SolidWorks (Dassault Systems, Waltham, MA). Computational fluid dynamic (CFD) simulation studies of the assembly were conducted in SolidWorks’ Flow Simulation package. Boundary conditions of inlet volumetric flow rate (0.5185 cm^3^/s for 4 dyne/cm^2^, 1.0370 cm^3^/s for 8 dyne/cm^2^, and 1.556 cm^3^/s for 12 dyne/cm^2^) and outlet pressure (3 mmHg) were assigned to the bioreactor assembly. A user-defined liquid representing CCM at 37°C was defined (density: 1.007 g/mL, dynamic viscosity: 0.0072 dyne*s/cm^2^, specific heat: 4.2 x 10^3^ J/Kg*K). As the perfusing media contained no cells or serum, it was characterized as a Newtonian liquid. Other assumptions and conditions included adiabatic wall thermal conditions, laminar and turbulent flow, gravity, and a PMMA surface roughness of 0.12 μm. Settings allowed for a high level of global and local mesh refinement as the simulation iteratively sought to reach convergence of velocity, flow rate, and shear stress calculations. 346,307 fluid cells described the study’s fluid flow. Results were displayed on a shear stress gradient heatmap. From the fluid shear stress heatmap, the surface area described by a target shear stress ± 1 dyne/cm^2^ was calculated. Velocity vector lines, in CFD simulations and the assembled device, were inspected to assess laminar versus turbulent flow.

### WSS Conditioning

Fluid shear stress was imposed in a similar manner as previously published work (28). Small-scale and large-scale bioreactors were manufactured in a similar manner. Each PPB was constructed from optically transparent polymethyl-methacrylate (PMMA; McMaster-Carr, Atlanta, GA) milled on an OM2 Haas computer numerical control machine (CNC, Haas Automation Inc, Oxnard, CA). Photopolymer (Digital ABS Plus, Stratasys, Eden Prairie, MN) gaskets printed on a J750 3D Printer (Stratasys) were placed between PMMA plates to establish individual channel height, while 3D-printed ports (VeroClear, Stratasys) distributed or collected fluid flow at the channel extremes. The PPB assembly includes two acrylic plates milled to a uniform size enclosed around the 3D-printed gasket (Digital ABS Plus). The assembly is completed by inserting translucent 3D-printed ports (VeroClear) into cutouts on the top acrylic plates. Individual components and luer lock connectors are bonded together with medical-grade epoxy (Henkel, Rocky Hill, CT). Each component is sequentially washed in detergent (Liquinox, Alconox Inc, White Plains, NY), 91% isopropyl alcohol, and DI water baths and dried in a laminar flow hood. Final assembly and bonding are carried out in the same laminar flow hood. After sterilization *via* ethylene oxide exposure or autoclaving, the PPB and flow-loop were handled within a tissue culture hood.

After three PBS washes to remove any remaining particulate and the PMMA was coated with human plasma fibronectin at a concentration of 10 µg/mL overnight (Gibco). Then, passage 4 hBM-MSC (PDL 17.5) or hAD-MSC (PDL 21.8) were loaded to a target concentration of 30,000 cells/cm^2^ (3.6 X 10^6^ MSC per PPB) and allowed to adhere to the bottom surface of conditioning chambers over a three-hour period. The optically transparent PMMA plates allowed for inspection of MSC at each step. Seeded bioreactors were coupled to a MasterFlex L/S Series Peristaltic Pump (Cole Parmer, Vernon Hills, IL) and continuously perfused in a unidirectional manner for three hours at a WSS of 4, 8, or 12 dyne/cm^2^. Requisite pump flow rate was calculated using the equation $\tau_{W}=-\mu \frac{du}{dy}$ , where μ is the fluid dynamic viscosity of CCM, *u* is the linear velocity of fluid flow, and *y* the distance from the channel’s boundary. After conditioning, the adherent cell population was washed with PBS and detached from the culture plastic using TrypLE Express.

The quantity and viability of MSC collected from each bioreactor was assessed with a NucleoCounter NC-200 using Via1 cassettes (Chemometec, Denmark). Static MSC cultured in filtered T-225 tissue culture plastic flasks (Nunc) were plated and harvested in parallel with the bioreactors. PPB were inspected by phase contrast light microscopy at each stage and after harvest.

### Measurements of PGE_2_ and IDO Production

Following WSS conditioning, cells were suspended at a concentration of 200,000/mL in CCM and dispensed as 1 mL per well (200,000 cells) into 6-well dishes that were then incubated for 18 hrs before the conditioned media was collected. PGE_2_ and IDO1 secreted by MSC into the culture medium were quantified using ELISA kits (Cayman Chemical, Ann Arbor, MI) in accordance with the manufacturer’s guidelines. Standard curves were processed in parallel for individual replicates. The resulting concentrations of PGE_2_ and IDO1 were calculated using regression analysis.

### Flow Cytometry

Suspended MSC samples were identified using a pre-mixed antibody panel of CD31, CD34, CD4, CD73, CD90, CD105, and CD146 (DURAClone SC Mesenchymal Stem Cell Panel, Beckman Coulter, Indianapolis, IN) as established by The International Society for Cellular Therapy as a minimal criteria (38). Data was acquired using an LSR II flow cytometer (BD Biosciences) and analyzed *via* Kaluza software (Beckman Coulter). Results are reported as a percentage of MSC expressing a given surface marker.

### Splenocyte Activation

Splenocyte isolation was performed as previously described (28, 34, 36, 39). After harvesting a fresh spleen from male Sprague Dawley rats under anesthesia, the organ was morselized using a 70 μm mesh filter. The collected material was suspended in ice cold PBS and centrifuged at 400 x *g* for 8 minutes. The supernatant was disposed of and the sample re-suspended in 10 mL red blood cell lysis buffer (Sigma-Aldrich, St. Louis, MO), undergoing perturbation for 5 min while still on ice. The sample was diluted with PBS and re-centrifuged at 400 x *g* for 8 min. The supernatant was disposed of and the pellet was suspended in phenol red-free RPMI with 10% FBS. The splenocytes were quantified and their viability assessed using a NucleoCounter NC-200. Splenocytes (2x10^6^ cells/mL) were left inactivated or activated with lipopolysaccharide (LPS) or concanavalin A (ConA). MSC-splenocyte cocultures were plated in wells at ratios of 1:80 or 1:20 (MSC : Splenocyte) for LPS and ConA cocultures, respectively. Culture supernatants were collected at 24 hrs after LPS administration or 48 hrs after ConA administration. The samples were analyzed using TNF-α or IFN-γ ELISA kits (Abnova, Taipei, Taiwan) following manufacturer’s guidelines. The hBM-MSC cell line tested was the same used in previous sections.

### RNA Extraction and RNA Analysis

Aliquots of 1 million cells were pelleted and snap-frozen in LN_2_ and submitted to Cancer Genomics Center core facility at The University of Texas Health Science Center at Houston (CPRIT RP180734). Total RNA was exacted by RNeasy Mini Kit (Qiagen, Hilden, Germany) and quality-checked using Agilent RNA 6000 Pico kit by Agilent Bioanalyzer 2100 (Agilent Technologies, Santa Clara, USA). All samples used in this study had an RNA integrity number greater than 7 and were subsequently used for library preparation. rRNA of 400ng total RNA were depleted with NEBNext rRNA Depletion Kit (New England Biolabs, Ipswich, MA) following the manufacturer’s instructions. The RNAs with more than 70nts were selected for preparation with NEBNext Ultra II Directional RNA Library Prep Kit for Illumina (New England Biolabs, Ipswich, MA) and NEBNext Multiplex Oligos for Illumina (New England Biolabs) following the manufacturer’s instructions. The quality of the final libraries was examined using Agilent High Sensitive DNA Kit by Agilent Bioanalyzer 2100 (Agilent Technologies), and the library concentrations were determined by qPCR using Collibri Library Quantification kit (Thermo Fisher Scientific). The libraries were pooled evenly and analyzed using paired-end 75-cycle sequencing on an Illumina NextSeq 550 System (Illumina, Inc., San Diego, CA, USA) using High Output Kit v2.5 (Illumina, Inc.).

All processing was done using Galaxy (40). Sequence files were processed using fastp to remove adapter sequences (41). HISAT2 was then used to align sequences to the hg38 canonical sequence (42). The alignment files were then sorted and merged using Samtools (43) and evaluated for quality using QualiMap for BamQC (44). A count file was then generated using featureCounts (45) which was then used for differential gene expression analysis using DeSeq2 (46). The resulting normalized counts were then used to determine the abundance of specific transcripts of interest.

The relevant data sets are available from the sequence read archive (SRA) as BioProject PRJNA814337.

### Statistical Analysis

Statistical analyses were conducted for independent replicates of 3 or more using two-tailed t-tests or one-way ANOVA with Tukey *post-hoc* analyses for multiple comparisons. A minimum significance level of 5% was used. Each mean is presented with the standard deviation and number of independent experiments (n). Unless stated otherwise, samples from a single bioreactor unit represent a single biological replicate. When appropriate, percent or fold difference from the control is presented. Graphpad Prism software (GraphPad, San Diego, CA) was used for statistical analysis.

## Results

### The MSC-Conditioning System Facilitates Uniform Fluid Dynamics

**Figure 1:**
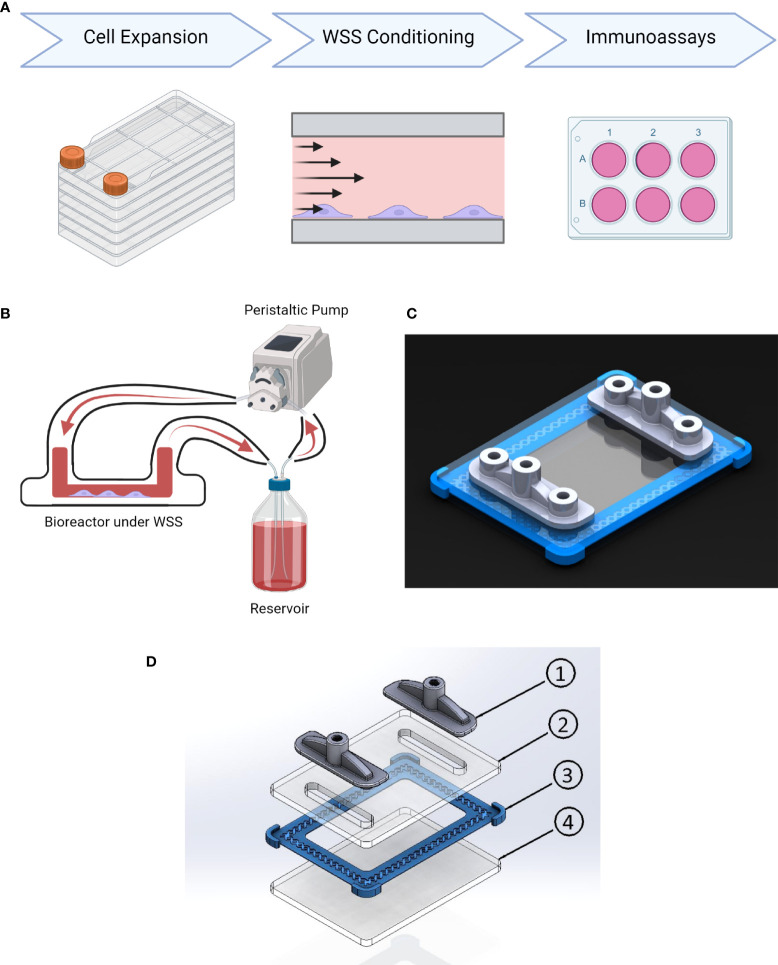
Conditioning platform design and procedure. **(A)** Experimental timeline. MSC undergo cell expansion in large volume tissue culture plastic under xeno-free conditions using cGMP-comparable methods and reagents. Next WSS conditioning is performed on adherent MSC in the parallel plate bioreactor at 4, 8, or 12 dyne/cm^2^ for 3 hrs. The resulting cells are then immunoassayed for their ability to modulate inflammation in a panel of assays. **(B)** Schematic of platform configuration, including PPB, media reservoir, and peristaltic pump. **(C)** Rendering of parallel-plate bioreactor displaying transparent PMMA plates fabricated using a CNC mill, 3D-printed gasket (blue), and 3D-printed inlet/outlet manifolds with sampling ports (grey). **(D)** Schematic of parallel plate bioreactor: (1) 3D Printed Inlet/Outlet Ports, (2) PMMA Top Plate, (3) 3D Printed Gasket, (4) PMMA Base Plate.

We designed, as part of a larger cell culture and validation pipeline, a scalable device to impose accurate WSS within a closed-loop system (**Figure 1**). The fluid dynamics of the resulting PPB and its ports were studied with computational simulations across a range of flow rates and operating conditions to generate continuous and consistent WSS. As the port geometry is the major influence on channel fluid dynamics, we iteratively designed and evaluated various geometries.

**Figure 2:**
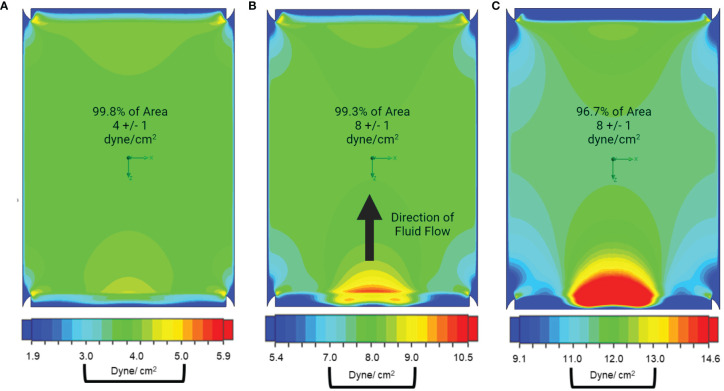
Our parallel plate bioreactor geometry facilitates uniform fluid dynamics and shear stress. Pseudocolor heatmaps of shear stress distribution across the growth surface of the bioreactor were generated using computer modeling. In this presentation, the fluid is flowing towards the top of the page and areas of low shear are presented in shades of blue, high shear is presented in shades of red, while the targeted shear +/- 1 dyne/cm^2^ are presented in shades of green. **(A)** When the operating conditions for 4 dyne/cm^2^ are simulated, we find that 99.8% of the surface area is between 3-5 dyne/cm^2^. **(B)** When the system is modeled at 8 dyne/cm^2^ 99.3% of channel surface area is within target wall shear stress and **(C)** 96.7% of channel surface area is within target when the operating conditions for 12 dyne/cm^2^ are simulated.

The final design was used for simulations to generate a shear stress gradient heatmap (**Figure 2**). From each heatmap, the surface area withstanding appropriate WSS magnitudes within ± 1 dyne/cm^2^ of the target magnitude was calculated. When using a calibrated pump with precise flow rate control, we predicted 99.8% of the surface is within the target range at 4 ± 1 dyne/cm^2^ conditions, 99.3% at 8 ± 1 dyne/cm^2^ conditions, and 96.67% at 12 ± 1 dyne/cm^2^ conditions. Little to no turbulent flow was produced by the entrance and exit geometries, regardless of flow rate. These simulations informed the construction of our final PPB used for uniform MSC mechanotransduction, illustrated in **Figure 1C**. The lack of turbulent flow was confirmed with dye-injection studies and visual inspection through the optically transparent PPB channels. Small-scale (20 cm^2^) and large-scale (120 cm^2^) PPB were manufactured for characterization at different experimental stages.

### Fluid Shear Stress Affects Cellular Viability and Attachment

To assess cellular viability and harvest yield, we plated hAD-MSC or hBM-MSC within chambers of our small-scale bioreactor or conventional tissue culture flasks. Adherent cells in a PPB were exposed to a range of fluid shear stress (4, 8, or 12 dyne/cm^2^) mimicking physiological conditions for 3 hrs, while the tissue culture flasks were left static (0 dyne/cm^2^). Our previous mechanotransduction studies, where a 3 hr conditioning period resulted in consequential functional enhancement, led us to use this schedule (28, 34).

**Figure 3:**
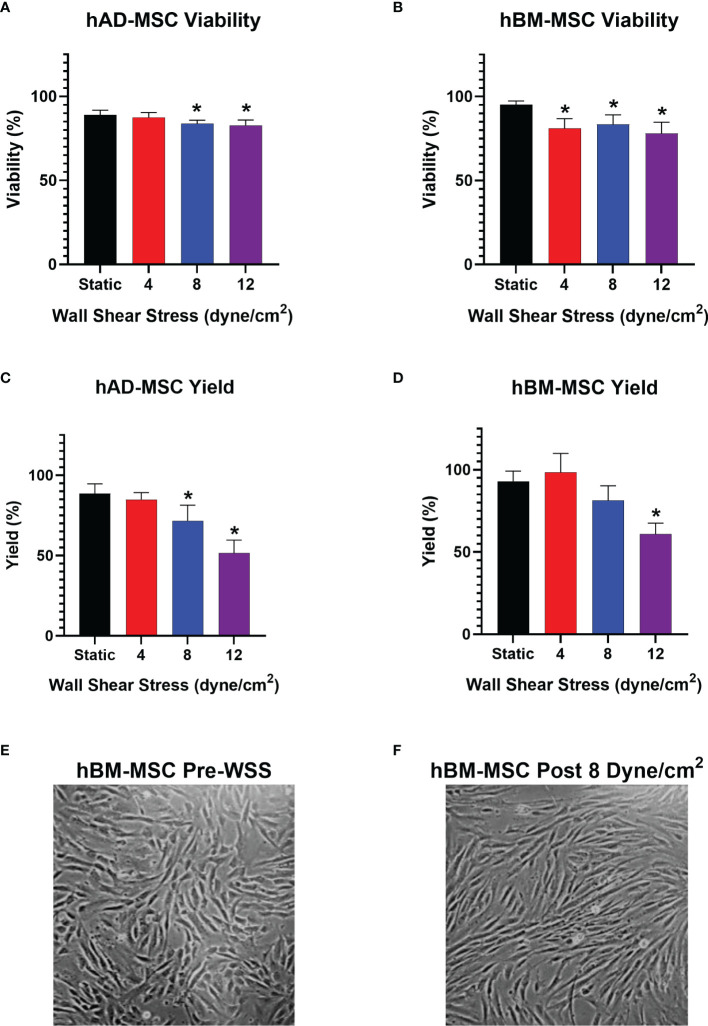
WSS-exposure impacts cellular yield, without affecting MSC viability. **(A)** Viability of harvested hAD-MSC after exposure to 4, 8, or 12 dyne/cm^2^ compared to static control culture (n=5 independent experiments; one-way ANOVA with Tukey’s multiple comparison test, *p<0.05 versus static group). Data represent Mean ± SD. **(B)** Viability of harvested hBM-MSC after exposure to 4, 8, or 12 dyne/cm^2^ (n=5 independent experiments for all groups, except n=4 in static group; one-way ANOVA with Tukey’s multiple comparison test, *p<0.05 versus static group). **(C)** Percent yield of hAD-MSC collected after exposure to 4, 8, or 12 dyne/cm^2^ compared to static control culture (n=5 independent experiments for all groups, except n=4 in 4 dyne/cm^2^ group; one-way ANOVA with Tukey’s multiple comparison test, *p<0.05 versus static group). **(D)** Percent yield of hBM-MSC collected after exposure to 4, 8, or 12 dyne/cm^2^ compared to static control culture (n=5 independent experiments for all groups, except n=4 in 4 dyne/cm^2^ group; one-way ANOVA with Tukey’s multiple comparison test, *p<0.05 versus static group). **(E, F)** Phase microscopy image (10x) taken 3 hrs after injecting hBM-MSC into fibronectin-coated bioreactor and after exposure to 8 dyne/cm^2^ for 3 hrs, respectively.

The experiment was first conducted with hAD-MSC, which showed a small but significant impact on viability after WSS exposure at 8 and 12 dyne/cm^2^ (**Figure 3A**). The cellular viability at 0 dyne/cm^2^ and 8 dyne/cm^2^ were 89.1% ± 2.7% and 83.9% ± 2.0%, respectively (*p*=0.0408). 12 dyne/cm^2^ conditions decreased viability to 82.7% ± 3.2% (*p*=0.0119 versus static).

The effect of WSS on hAD-MSC yield was more profound at high WSS magnitudes (**Figure 3C**). The percentage of cells recovered following harvest from static and 4 dyne/cm^2^ cultures were similar at 88.64% ± 6.0% and 84.8% ± 4.4%, respectively (*p*=0.8703). 8 dyne/cm^2^ and 12 dyne/cm^2^ conditions significantly decreased yield to 71.7% ± 9.7% and 51.5% ± 8.1%, respectively (*p*=0.0152 and *p*=<0.0001 versus static group, respectively).

We repeated the experiment with hBM-MSC. The viability of hBM-MSC cultured statically was 95.2% ± 2.2%, while 4 and 8 dyne/cm^2^ conditions decreased viability to 81.0% ± 5.9% and 83.5% ± 5.6%, respectively (*p*= 0.0077 and *p*=0.0299 versus static, respectively) (**Figure 3B**). 12 dyne/cm^2^ resulted in the greatest viability decrease relative to static culture (78.1% ± 6.6%, *p*=0.0017).

hBM-MSC exhibited a yield reduction similar to hAD-MSC at higher WSS. Static and 4 dyne/cm^2^ culture conditions resulted in yields of 93.0% ± 6.2% and 98.4% ± 11.5%, respectively. Applying 8 dyne/cm^2^ led to a harvest of 81.4% ± 8.9% (*p*= 0.1672 versus static). After exposure to 12 dyne/cm^2^, there was a significant drop in yield relative to static cultures (60.9% ± 6.7%, *p*=0.0001) (**Figure 3D**).

Microscopic examination of hBM-MSC after a 3 hr seeding period in the bioreactors shows cells readily adhering in a monolayer (**Figure 3E**). Re-examination of the same cell population after subsequent conditioning at 8 dyne/cm^2^ revealed slight changes in cell morphology (**Figure 3F**).

### WSS Induces Immunomodulatory Mechanisms in MSC

**Figure 4:**
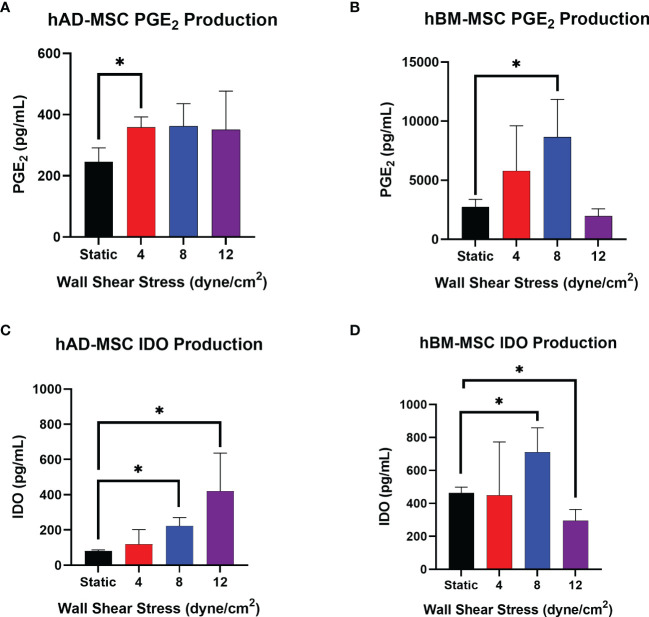
Select WSS-exposure enhances production of PGE_2_ and IDO by hAD-MSC and hBM-MSC. **(A, B)** The accumulation of PGE_2_ in conditioned media from hAD-MSC and hBM-MSC cultures, respectively, plated at 200,000/mL, was assayed after an 18 hr incubation using ELISA (n=3 independent experiments; unpaired t-test, *p<0.05). Data represent Mean ± SD. **(C, D)** The concentration of IDO in conditioned media from hAD-MSC and hBM-MSC cultures plated at 200,000/mL was determined after 18 hrs incubation using ELISA (n=3 independent experiments; unpaired t-test, *p<0.05).

Conditioned media from hAD-MSC exposed to 0, 4, 8, and 12 dyne/cm^2^ was generated by plating 200,000 cells per well into a 6 well plate and incubating for 18hrs to measure the production and accumulation of PGE_2_ and IDO1. Of the WSS magnitudes tested, 4 dyne/cm^2^ significantly increased PGE_2_ production relative to static culture (**Figure 4A**, 358.2 ± 34.2 pg/mL versus 245.9 ± 45.1 pg/mL, *p*=0.0264). The PGE_2_ concentrations of 8 and 12 dyne/cm^2^ cultures (362.5 ± 73.3 pg/mL and 350.9 ± 125.6 pg/mL, respectively) were not significantly different from the static group (*p*=0.0788 and *p*=0.2444, respectively).

hAD-MSC IDO1 production significantly increased after exposure to 8 dyne/cm^2^ from 80.8 ± 6.0 pg/mL to 222.9 ± 47.2 pg/mL (**Figure 4C**, *p*=0.0016). The average IDO1 concentration in 12 dyne/cm^2^ wells was measured at 420.5 ± 215.9 pg/mL, which also reached significance (*p=*0.0225).

Relative to the hAD-MSC line, the naïve hBM-MSC line produced more PGE_2_ (**Figures 4A, B**). Also, the hBM-MSC line exhibited a greater response to fluid shear stress exposure as reflected by a larger fold-change. PGE_2_ secretion increased after conditioning hBM-MSC with 8 dyne/cm^2^ from 2731.3 ± 645.4 pg/mL to 8657.7 ± 3189.0 pg/mL (**Figure 4B**, *p*=0.0135).

Mechanotransduction with 8 dyne/cm^2^ significantly increased hBM-MSC production of IDO1 from 462.8 ± 35.6 pg/mL to 710.0 ± 148.5 pg/mL (**Figure 4D**, *p*=0.0485). Conditioning with 12 dyne/cm^2^ actually decreased IDO1 with a concentration of 295.9 ± 67.4 pg/mL measured (*p*=0.0192). As with PGE_2_, the hBM-MSC line produced more IDO1 at baseline than the hAD-MSC line (**Figures 4C, D**).

### Wall-Shear Stress Does Not Alter MSC Phenotype

The previous section shows mechanotransduction enhances anti-inflammatory protein production in a shear-dependent manner. This data, in combination with viability and cellular yield findings, led us to select 8 dyne/cm^2^ as an optimal WSS for further evaluation. The PPB was scaled to create a 120 cm^2^ surface area to facilitate larger cell numbers for the following experiments (unless otherwise noted). Additionally, we focused on the use of a cGMP-simulated cell line to facilitate future translational applications.

We evaluated the expression of classic MSC markers CD73, CD90, CD105, and CD146 on mechanotransduced hBM-MSC using a pre-mixed antibody panel (39). An established reference BM-MSC cell line (hBMMSC 5204, PDL 12.9) and non-mechanotransduced hBM-MSC served as controls (Roo205 Static). WSS-MSC were negative for the hematopoietic lineage markers CD31, CD34, and CD45 (**Table 1**). Less than 1% of the WSS-MSC population expressed CD31, CD34, and CD45. These findings were comparable to the external reference hBM-MSC, which expressed CD31, CD34, and CD45 at rates of 0.69%, 0.10%, and 3.28%, respectively. Greater than 98% of WSS-MSC expressed the positive hMSC immunophenotypic markers CD73, CD90, and CD105. Over 87% of the hBM-MSC expressed CD146. At least 10,000 events were evaluated for each population.

**Table 1:** hBM-MSC cell surface marker expression is unaltered after mechanotransduction.

Table of MSC Markers (% positive)							
	Negative Markers			Positive Markers			
	CD31	CD34	CD45	CD73	CD90	CD105	CD146
Reference hBM-MSC	0.69	0.10	3.28	99.65	99.64	99.56	97.75
Roo205p4 (static culture)	0.14	0.03	0.46	99.69	99.68	99.55	93.75
Roo205p4 (small PPB) 8.1	0.48	0.00	0.12	98.76	98.93	98.29	90.08
Roo205p4 (small PPB) 8.2	0.29	0.15	0.00	99.34	99.39	98.54	87.57
Roo205p4 (Full PPB) 8.1	0.18	0.07	0.11	99.72	99.61	99.15	89.11
Roo205p4 (Full PPB) 8.2	0.35	0.05	0.02	99.08	99.00	98.44	88.12

The phenotype of each cell population was determined using a panel of markers used to identify and define MSCs. Typical expression of the markers is provided by an external bone marrow MSC. The experimental populations consist of the non-sheared control group, Roo205p4 (static culture), the replicate cultures treated with WSS generated using the initial small scale PPB, Roo205p4 (small PPB) 8.1 and 8.2, and the replicate cultures treated with WSS using the large scale PPB, Roo205p4 (large PPB) 8.1 and 8.2.

### Mechanotransduced hBM-MSC Suppress Inflammatory Cytokine Release From Activated Splenocytes *In Vitro*

Similar to previously published work, hBM-MSC conditioned with 8 dyne/cm^2^ were studied using a superantigen-activated splenocyte coculture assay, a functional assay that simulates some of the complex MSC-immune effector cell interactions expected *in vivo* (28, 34, 36, 39). Two independent large-scale bioreactors were run side-by-side to compare performance (Roo205 8.1 and Roo205 8.2). The resulting WSS-MSC and splenocyte cocultures were performed in triplicate, which are presented as replicates from each PPB. Previous studies influenced the choice of MSC:splenocyte ratio for the LPS and ConA-stimulated cultures (28, 36, 47, 48).

**Figure 5:**
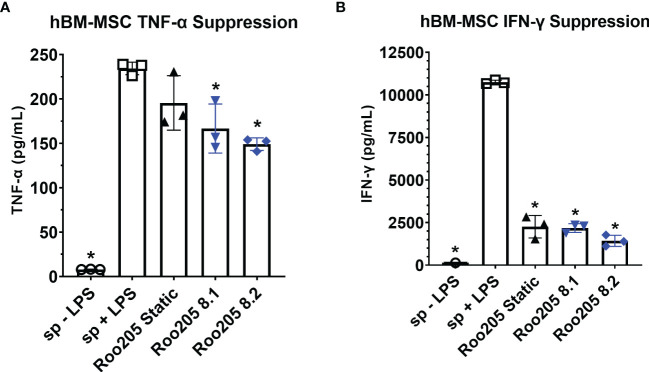
WSS-conditioned hBM-MSC suppress activated splenocyte cytokine production. **(A)** The TNF-α concentration produced by LPS-activated splenocyte in co-culture with MSC at a ratio of 1 MSC: 20 splenocytes, after 24 hrs incubation determined using a rat-specific ELISA (n=3 independent experiments; one-way ANOVA with Tukey’s multiple comparison test, *p<0.05, LPS-activated splenocytes versus other groups). Data represent Mean ± SD. **(B)** The IFN-γ concentration produced by ConA-activated splenocyte in co-culture with MSC at a ratio of 1:80 MSC to splenocyte, after 48 hrs incubation determined using a rat specific ELISA (n=3 independent experiments; one-way ANOVA with Tukey’s multiple comparison test, *p<0.05, ConA-activated splenocytes versus other groups). Data represent Mean ± SD.

MSC were cocultured with LPS-stimulated splenocytes at a ratio of 1:80 and incubated for 24 hrs before the supernatant was collected. WSS-MSC from the two PPB significantly reduced TNF-α production by LPS-stimulated splenocyte from 234.4 ± 7.0 pg/mL to 166.7 ± 27.6 pg/mL and 149.2 ± 7.1 pg/mL (**Figure 5A**, *p*=0.0095 and *p*=0.0019, respectively). Coculturing with Static-MSC did not significantly reduce the average supernatant concentration (195.5 ± 30.6 pg/mL, *p*=0.1643 versus activated-splenocyte).

MSC were cocultured with ConA-stimulated splenocytes at a ratio of 1:20 and incubated for 48 hrs before the supernatant was collected. Both, WSS-MSC and Static-MSC significantly reduced IFN- γ production (**Figure 5B**). Static-MSC decreased the measured concentration from 10,749.0 ± 111.7 pg/mL in activated splenocyte cultures to 2260.4 ± 660.8 pg/mL (*p*<0.0001). The IFN- γ concentrations in Roo205 8.1 and Roo205 8.2 cocultures were measured at 2176.7 ± 254.4 pg/mL and 1427.8 ± 329.8 pg/mL, respectively (*p*<0.0001 versus activated-splenocyte, for both groups).

### Mechanotransduction Results in Differential Gene Expression of Immunomodulatory Factors

**Figure 6:**
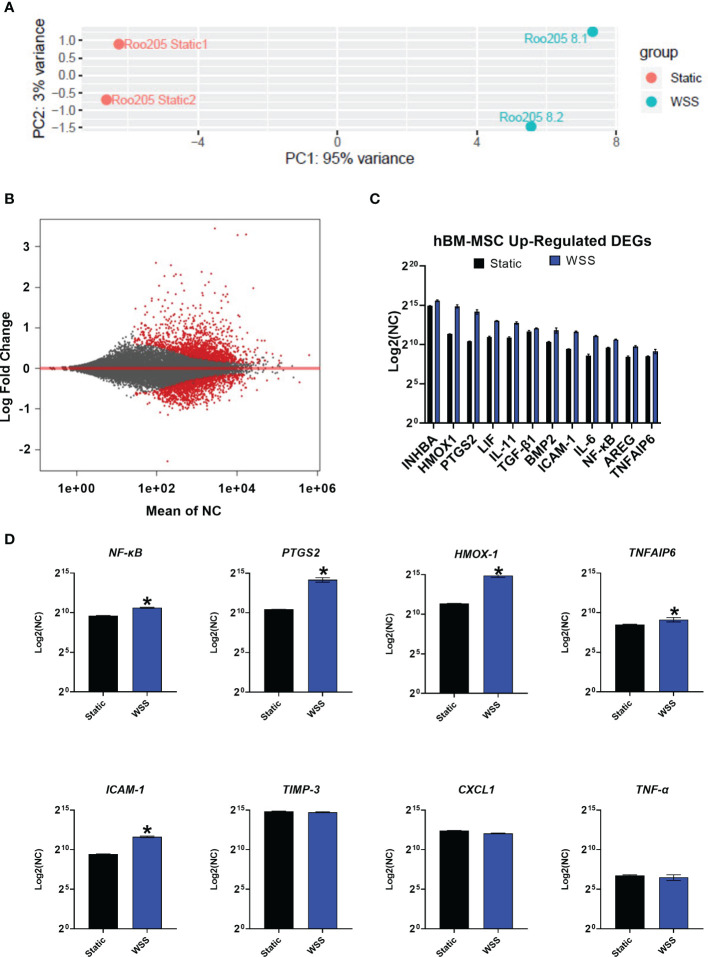
Transcriptome analysis of mechanotransduced hBM-MSC shows enhanced expression of immune mediators. **(A)** Principal component analysis of conventionally cultured (Static) and 8 dyne/cm^2^ conditioned hBM-MSC (WSS) (n=2). Samples are plotted by variance across the two primary principal components (PC1, PC2). **(B)** MA Plot depicting distribution of differentially expressed genes (DEG, in red) plotted by the mean normalized count (NC) against the log fold change. **(C)** A curated list of WSS-induced, up-regulated differentially expressed genes. Y-axis depicts NC by DESeq2 (n=2). **(D)** Expression of established inflammatory mediators. Y-axis depicts NC (n=2, *p-adj<0.05). *INHBA*, Inhibin Subunit Beta A; *HMOX1*, Heme oxygenase 1; *PTGS2*, Prostaglandin-Endoperoxide Synthase 2; *LIF*, Leukemia Inhibitory Factor; *IL-11*, Interleukin 11; *TGF-β1*, Transforming Growth Factor Beta 1; *BMP2*, Bone Morphogenetic Protein 2; *ICAM-1*, Intracellular Adhesion Molecule 1; *IL-6*, Interleukin 6; *NF-κB*, Nuclear Factor Kappa B; *AREG*, Amphiregulin; *TNFAIP6*, Tumor Necrosis Factor-Inducible Protein 6; *TIMP-3*, Tissue Inhibitor of Metalloproteinase 3; *CXCL1*, C-X-C Motif Chemokine Ligand 1; *TNF-α*, Tumor Necrosis Factor Alpha.

We performed whole exome sequencing on duplicate cultures of hBM-MSC conditioned with 8 dyne/cm^2^ followed by differential gene expression analysis comparing them to static cultures. Principal component analysis (PCA) of the samples displays that WSS-exposure resulted in a much larger distribution of differentially expressed genes (PC1) compared with the variation between replicates (PC2) (**Figure 6A**). After biomechanical pre-conditioning, 988 genes exhibited differential expression (**Figure 6B**, p-adj < 0.05) (49, 50). 646 of these differentially expressed genes were upregulated (65.4%). Differential gene expression analysis demonstrated 313 genes with a p-adj < 0.01. This limited subset of transcripts features a number of immunomodulatory genes.

We evaluated expression of immunologically influential genes often discussed as therapeutic mechanisms for MSC: *NF-κB, PTGS2, HMOX-1, TNFAIP6*, *ICAM-1, TIMP-3, TNF-α*, and *CXCL1* (**Figure 6D**). The genes *NF-κB, PTGS2, HMOX-1, TNFAIP6*, and *ICAM-1* were significantly upregulated (p-adj<0.05), while *TIMP-3* was similarly expressed by the WSS-MSC and Static-MSC (p-adj= 0.7246). As expected, genes for typical proinflammatory cytokines, such as TNF-α and CXCL1, were not affected by WSS and detected at low levels (p-adj = 0.8945 and 0.7897, respectively).

Finally, we screened the list of up-regulated, differentially expressed genes (p-adj <0.05) for commonly reported immune modulators. Genes of interest included *INHB1, VEGFA, LIF, IL-11, SERPINB2, TGF-β1, BMP2, IL-6, MT1X, AREG, ATF3*, and *FGF11* (**Figure 6C**).

## Discussion

This study describes a set of experiments that are proof-of-concept and the first steps in realizing a clinically translatable mechanotransduction strategy. We designed a scaled parallel plate bioreactor to exert uniform WSS on adherent cells in culture and then performed a series of experiments to evaluate changes in MSC immunomodulatory potential after conditioning with fluid shear stress. We utilized computer modeling to generate PPB where >96% of the surface area was within 1 dyne/cm^2^ of our target WSS value. After manufacturing our PPB, we found that human MSC from two different tissues (adipose tissue and bone marrow) exhibited high viability across the WSS range studied, although 12 dyne/cm^2^ resulted in a lower cellular yield. The WSS mechanotransduction increased expression of PGE_2_ and IDO1 consistently at 8 dyne/cm^2^, similar to smaller scale experiments utilizing multiple cell lines (28, 34). 8 dyne/cm^2^ was selected as an optimal WSS for additional characterization of hBM-MSC. We found that WSS increased the ability to reduce TNF-α production by activated splenocytes compared to static hBM-MSC cultures. All groups tested drastically reduced IFN-γ production by ConA-stimulated splenocytes. RNAseq analysis found a subset of differentially expressed genes that contained known therapeutic mechanisms of action by MSC. Over the course of our mechanotransduction studies, we’ve used many PPB iterations, some of which were used in studies demonstrating increased potency in multiple MSC cell lines (28, 34). This particular PPB model conditions an exponentially larger cell population than previously attempted and is the first step in scaling the concept. During its development and characterization, we used over 25 devices. The consistent maturation of WSS-MSC products validates our novel, scalable platform.

The concept of inflammatory licensing pervades the field of MSC biology. Previous studies by our group and others have evaluated the effects of inflammatory cytokines (4, 12, 14–16, 21, 24, 39, 51), hypoxia (23, 24), serum starvation (25, 26), and numerous other culture manipulations. Mechanotransduction of MSC is particularly interesting for several reasons. First, MSC are abruptly exposed to vascular fluid dynamics when migrating or when infused intravenously or intra-arterially (6, 29, 31, 33, 52–55). Previous studies evaluating the response of numerous cell lines indicate that MSC respond to this shear stress by expressing a number of potentially therapeutic mechanisms (28, 31, 32, 34). Finally, the application of WSS does not require the use of potentially dangerous culture additives that may complicate future clinical applications (2, 8, 11, 12). A WSS-conditioning strategy is an intrinsically simpler approach to increasing potency than genetic manipulation or molecular licensing, necessitating minimal adjustments to a cell therapy manufacturing pipeline and reagent list.

In previous work, our group found that mechanotransduced hBM-MSC were primed towards anti-inflammatory activity based on WSS-dependent focal adhesion kinase (FAK) signaling and described the resulting functional potentiation (28, 32, 34). Specifically, fluid shear stress activates the FAK/NF-κB signaling pathway to enhance production of proteins implicated in anti-inflammatory immune modulation, such as heme oxygenase-1 (HO-1) and PGE_2_ in three independent hBM-MSC donor cell lines (34). Finally, an *in vivo* study evaluating five hBM-MSC cell lines demonstrated the increased potency of WSS-MSC in a rat TBI model, finding that WSS-MSC decreased apoptotic and M1-type activated microglia after injury (28).

Given their increased *in-vivo* potency, WSS-MSC might reduce the cell dose needed to treat, thereby easing manufacturing burden and associated costs compared to naïve MSC. With this in mind, we redesigned the WSS platform used in our previous studies to create a scalable, clinically-translatable mechanotransduction device and replicate relevant characterization assays (28, 34).

A fully sterilizable closed-circuit flow loop containing a PPB, commercially available connectors, and tubing was engineered to facilitate sterile media transfer and sampling. The custom bioreactor was iteratively designed using CFD studies to apply reproducible, accurate fluid shear stress on adherent MSC populations. While the PPB’s components were individually fabricated for this study and hand-assembled, the components are compatible with large-scale injection molding and automated assembly processes. Furthermore, the bioreactor’s length can be increased to accommodate more surface area and cells without drastic alteration of the reactor fluid dynamics.

As MSC manufacturing has significant bottlenecks and real-world financial constraints, it is important to optimize the number of viable cells harvested from a PPB. Our composite results from hAD-MSC and hBM-MSC suggest a strong negative correlation between WSS and cellular yield, with a smaller effect size on viability. These findings agree with a great deal of literature concerning MSC biomanufacturing and the inverse relationship between flow rate and cellular attachment (8, 56, 57). The findings suggested precluding WSS magnitudes ≥12 dyne/cm^2^ due to diminishing returns.

Next, we assayed the changes of selected MSC therapeutic mechanisms and immunomodulatory activity after exposure to physiologically relevant shear stress magnitudes. PGE_2_ and IDO1 are two well described mechanisms by which MSC exert anti-inflammatory activity on various immune (4, 12, 28, 36, 58). PGE_2_, an eicosanoid with pleiotropic effects, is upregulated in response to many cytokines, mitogens, and pharmacological agents (12). The paracrine signaling molecule contributes to the resolution of neutrophil-mediated inflammation, attenuation of natural killer cell activity, suppression of pro-inflammatory macrophages, and inhibition of CD8+ T cells (5, 7, 10, 28). IDO1, classically secreted by MSC in response to IFN- γ stimulation, metabolizes tryptophan into kynurenine metabolites. The depletion of tryptophan induces CD8+ and CD4+ Th1 T cell anergy, suppresses allogeneic T-cell responses, and induces proliferation of T-regulatory cells (4, 59).

Fluid shear stress enhanced PGE_2_ and IDO secretion in two different MSC derivations, supporting the conservation of mechanotransduction effect after scaling. There was a magnitude-dependent relationship between shear stress and PGE_2_/IDO1 production that resulted in diminished returns at 12 dyne/cm^2^. Our preparations of hBM-MSC and hAD-MSC exhibited similar relative responses to WSS, although hBM-MSC produced higher amounts of PGE_2_ and IDO. This difference could be solely due to a limited number of samples assayed, and literature regarding PGE_2_ production by the two derivations is inconclusive (60–63). Increased PGE_2_ and IDO1 production, two soluble factors with established *in vivo* mechanisms of action, indicates that WSS-MSC exhibit some of the secretome patterns of licensed MSC (1, 2, 4, 5, 7, 12, 49, 58). These findings guided our selection of 8 dyne/cm^2^ and hBM-MSC for further investigation.

We evaluated the *in vitro* potency of hBM-MSC mechanotransduced with 8 dyne/cm^2^ *via* a cytokine suppression assay using antigen-stimulated primary rat splenocytes. The assay serves as an approximation of *in vivo* immune relations and includes the complexity of cell-to-cell interaction in a mixed leukocyte population (28). Cells of monocyte lineage are the prime producers of short-term TNF-α when stimulated with LPS activation (64). The secretion of TNF-α correlates with severity of inflammation and the innate immune system response (5, 11). In a similar set of experiments, ConA primarily activates T cells resulting in the accumulation of IFN- γ over a slightly longer period, thus cultures were sampled at 48 hrs. IFN-γ,classically associated with Th1 response and CD8+ T cell activation, served as a general indicator of the adaptive immune system (5).

Indeed, mechanotransduced hBM-MSC dampened TNF-α and IFN-γ production by activated splenocytes. In the case of TNF-α, the reduction in the WSS-MSC coculture was significant. Static-MSC contributed to a modest, but not statistically significant decrease. This enhanced suppression of TNF-α by WSS-MSC relative to Static-MSC is evidence of functional augmentation *via* mechanotransduction and successful scaling of the priming methodology. Both MSC groups significantly decreased IFN-γ concentrations, nearly to the level measured in unstimulated splenocyte cultures. Notably, little functional difference was observed between cells conditioned in independent bioreactors.

We carried out RNASeq on BM-MSC conditioned with 8 dyne/cm^2^ to elucidate the effect of mechanotransduction on the transcriptome of MSC. This sequencing provides an initial analysis of the WSS-MSC transcriptional signature. PCA and MA plots depict a robust effect with transcriptional patterns shifting after WSS conditioning.

Focused RNASeq analysis found the genes *NF-κB* and *PTGS2*, both commonly associated with immune modulation mechanisms (1, 3, 5, 9, 11, 49), increased after WSS. Activation of the NF-κB-COX2-PGE_2_ pathway is a recognized component of MSC potency, especially in regard to monocyte and T-cell inhibition (11, 34) and is a primary consequence of biomechanically stimulated FAK signaling (34). *PTGS2* encodes for cyclooxygenase-2, the rate-limiting enzyme in PGE_2_ production, and its increased expression further confirms the observed increase in PGE_2_ secretion.

Other known therapeutic mechanisms of MSC were increased including *TNFAIP6, ICAM-1*, and *HMOX-1*.*TNFAIP6*, or TNF-α inducible protein 6, encodes for TSG-6, a hyaluronan-binding protein that induces macrophage plasticity (65). Increased TSG-6 production is reported to ameliorate proinflammatory-driven neuroinflammation in stroke and lung injury models (65–67). The adhesion molecule ICAM-1 provides the means for direct cell-to-cell contact between MSC and immune effector cells. Upregulation of ICAM-1 is an indicator of enhanced MSC immunomodulatory capacity (49). Once engaged with monocytes or T cells, MSC can provide direct signals to induce apoptosis, cell cycle arrest, or class-switching switching (39, 49). *HMOX-1*, encoding the antioxidant HO-1, was also significantly upregulated after mechanotransduction. HO-1 is the rate-limiting enzyme in heme degradation (68). The stress-induced protein not only plays a critical role in oxidative stress protection and iron detoxification but has been shown to prevent allograft rejection and promote anti-inflammatory T cell responses through induction of IL-10 (51, 68). The upregulation of these genes should be studied in the context of disease-specific clinical applications.

Broader transcriptome analysis yielded a list of mechanically-stimulated, differentially expressed genes that are yet to be conclusively associated with mechanotransduction. This includes IL-6, leukemia inhibitory factor (LIF), and IL-11. These members of the IL-6 family, heavily regulated by NF-κB, were each differentially expressed after mechanical conditioning. These immune modulators provide neuroprotection after ischemic CNS injury by inhibiting microglia activation, regulating adaptive immune system tolerance, and influencing CD4+ T Cell lineage (49, 69–73). Members of the TGF-β family (Activin A, TGF-β1, and BMP2) were also differentially upregulated. This family is classically associated with injury resolution, anti-apoptotic signaling, enhanced T regulatory cell differentiation, microglia suppression, and CNS neurogenesis after excitotoxic neurodegeneration (7, 69, 74, 75). These findings highlight MSC’s role as soluble factor generators, exerting their immunomodulatory influence through a myriad of mechanisms (49, 50, 76). A broader analysis with more experimental groups is required to evaluate this further.

A limitation to this study was its use of a single donor for each MSC tissue derivation. The number of samples and replicates were limited by the number and size of PPBs available, as each reactor was custom built, assembled, sterilized, and tested for this study. As the bioreactors move towards a final production process and become more available, we will replicate and confirm our findings across additional tissue sources, donors, and production lots of MSCs. Based on previous work comparing independent donor cell lines, WSS enhances the protein production and immunomodulatory potential different cell lines to varying degrees, which must be further quantified (34). In this study, other protocol variables were not studied (e.g., time, waveform frequency, etc.) that could further optimize the effects of WSS on MSC potency. Also, more research must be done to evaluate the duration of the mechanotransduction effect, specifically concerning conservation after a freeze-thaw cycle to facilitate acute and sub-acute applications. While the *in-vitro* splenocyte assay demonstrated various WSS-MSC immunomodulatory properties, the cell therapy must be assessed in a disease-specific animal model for more generalizable findings. Finally, we anticipate that the system will require additional scaling of the bioreactor, increasing channel surface area and the number of channels per bioreactor, to accommodate larger human dosages (between 1-10 million cells/kg).

The application of fluid shear stress to an adherent MSC population within a PPB is a scalable preconditioning methodology that requires little manipulation beyond the addition of fluid dynamics, capable of being adapted and applied for future clinical MSC products. We assessed several critical parameters of cellular manufacturing including viability, yield, identity, and immunomodulatory potential after WSS conditioning. The findings of this characterization support the feasible manufacturing of biomechanically conditioned MSC. Future efforts should confirm the attributes of a standardized, mechanotransduced MSC therapy and reduction to phenotypic heterogeneity in other cell lines. Leveraging the responsiveness of MSC to biophysical cues might yield a novel licensing approach and enhanced therapy for inflammatory indications.

## Data Availability Statement

The data presented in the study are deposited in the sequence read archive (SRA) repository, BioProject PRJNA8143337, accession numbers SAMN26548527, SAMN26548528, SAMN26548529, and SAMN26548530.

## Author Contributions

Conception and design: MS, SO, BG, and CC. Collection and/or assembly of data: MS, SO, and KP. Data Analysis and interpretation: MS, SO, KP, BG, and CC. Manuscript writing: MS, SO, BG, and CC. Final approval of manuscript: MS, SO, BG, and CC.

## Funding

This study is supported by the Clare A Glassell Family Pediatric Surgery Research Fund; Alpha Omega Alpha Carolyn L. Kuckein Student Research Fellowship; NIH NINDS R21NS116302. The authors declare that this study also received funding from Cellvation. The funder was not involved in the study design, collection, analysis, interpretation of data, the writing of this article or the decision to submit it for publication.

## Conflict of Interest

SO has received research support from Athersys, CBR Systems, Hope Bio, Generate Life Sciences, Cellvation, and Biostage. CC has received research support from Athersys, CBR Systems, Hope Bio, Biostage, Generate Life Sciences, and Cellvation, and is on the Scientific Advisory Board of Cellvation and Generate Life Sciences. BG is on the Scientific Advisory Board of Cellvation.

The remaining authors declare that the research was conducted in the absence of any commercial or financial relationships that could be construed as a potential conflict of interest.

## Publisher’s Note

All claims expressed in this article are solely those of the authors and do not necessarily represent those of their affiliated organizations, or those of the publisher, the editors and the reviewers. Any product that may be evaluated in this article, or claim that may be made by its manufacturer, is not guaranteed or endorsed by the publisher.
