# Supplementary figures and images for: Enhancing Mesenchymal Stromal Cell Potency: Inflammatory Licensing via Mechanotransduction
Source: Front Immunol. 2022 Jul 6;13:874698. doi: 10.3389/fimmu.2022.874698 (PMC9297916; doi:10.3389/fimmu.2022.874698)

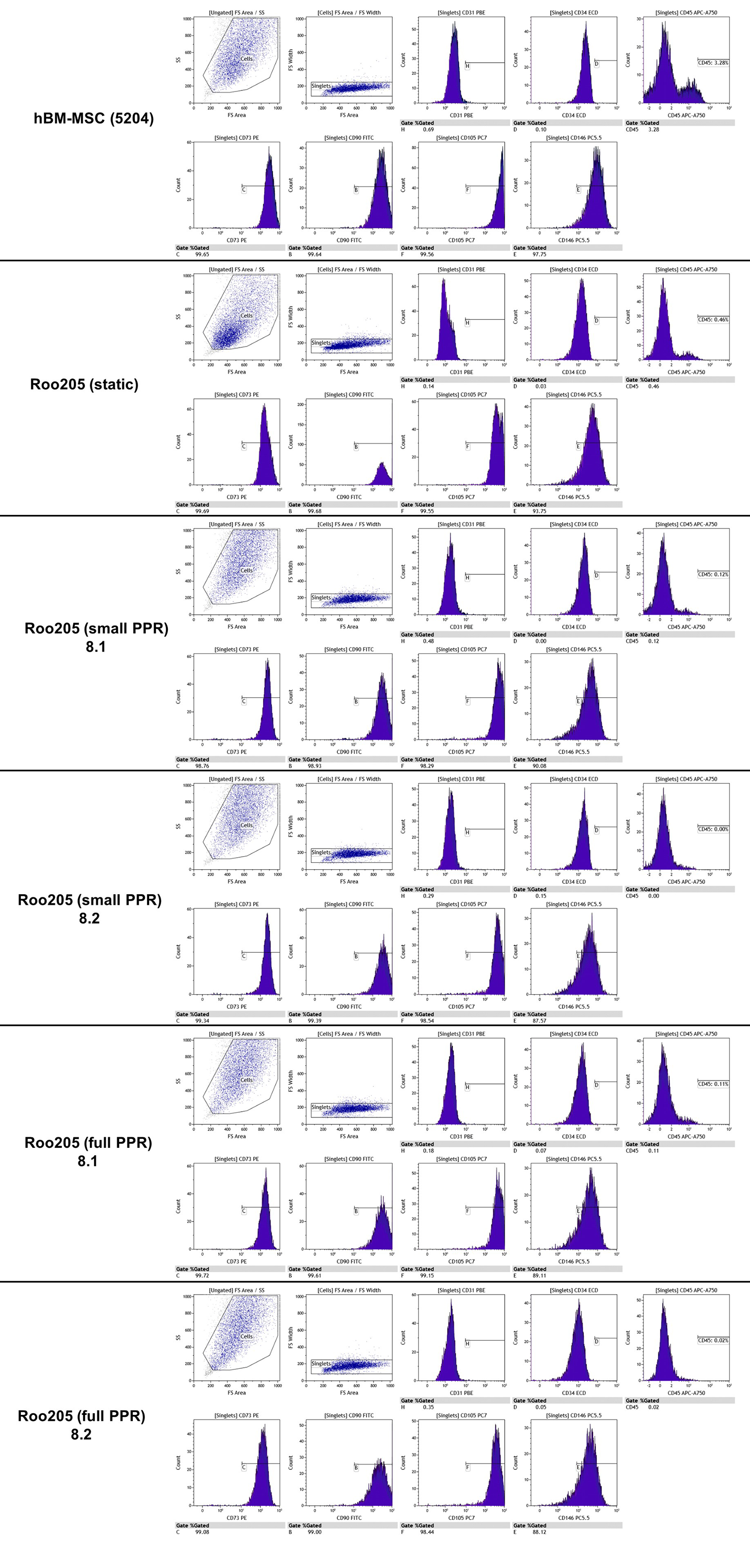

Supplement: Supplementary file 2 [file Image_1.tif]
